# Enhanced Efficacy of Some Antibiotics in Presence of Silver Nanoparticles Against Multidrug Resistant *Pseudomonas aeruginosa* Recovered From Burn Wound Infections

**DOI:** 10.3389/fmicb.2021.648560

**Published:** 2021-09-20

**Authors:** Maha A. Khalil, Gamal M. El Maghraby, Fatma I. Sonbol, Nanis G. Allam, Perihan S. Ateya, Sameh S. Ali

**Affiliations:** ^1^Biology Department, College of Science, Taif University, P.O. Box 11099, Taif 21944, Saudi Arabia; ^2^Botany and Microbiology Department, Faculty of Science, Tanta University, Tanta, 31527, Egypt; ^3^Pharmaceutical Technology Department, Faculty of Pharmacy, Tanta University, Tanta, 31527, Egypt; ^4^Pharmaceutical Microbiology Department, Faculty of Pharmacy, Tanta University, Tanta, 31527, Egypt; ^5^Biofuels Institute, School of the Environment and Safety Engineering, Jiangsu University, Zhenjiang, 212013, China

**Keywords:** *Pseudomonas aeruginosa*, drug resistance, burn wound infection, antimicrobial activity, nanoparticle-antibiotic combination, pharmaceutical formula

## Abstract

Burn wound infections with multidrug-resistant (MDR) bacteria are shown in many countries as severe widespread health threats. Consequently, attention has been devoted to new nanoparticle-based materials in the field of antimicrobial chemotherapy for burn wound infections. This study aimed to evaluate both *in vitro* and *in vivo* efficacies of nanoparticle–antibiotic combinations as new classes of materials subjected against MDR *Pseudomonas aeruginosa*. Out of 40 Gram-negative isolates, 23 *P. aeruginosa* were recovered from patients with burn wound infections attending different hospitals in Tanta, Egypt. The susceptibility test revealed that 95.7% of *P. aeruginosa* isolates were MDR with a high incidence of resistance against carbenicillin. Antibacterial activities of silver nanoparticles (Ag-NPs) against the isolates examined showed various inhibition zone diameters ranging from 11 to 17 mm. Strong synergistic efficacy of neomycin was reported in combination with Ag-NPs against MDR *P. aeruginosa* P_8_ and P_14_ isolates. The *in vivo* effectiveness of various pharmaceutical formulations prepared from a combination of neomycin antibiotic with Ag-NPs in the treatment of induced bacterially infected mice burns showed that maximum healing activity along with faster wound contraction reported with the combination of neomycin-Ag-NPs in the spray formulation. Generally, data indicated that incorporating Ag-NPs in combination with certain antibiotics may be a new, promising application for wound treatments, especially burns infected with MDR *P. aeruginosa*.

## Introduction

Burn wound infections are a significant cause of death in burn patients, particularly those caused by MDR pathogens such as *Pseudomonas aeruginosa*, methicillin-resistant *Staphylococcus aureus* (MRSA), and vancomycin-resistant Enterococci. These infections can result in healing failure and are also used as a predictor of predicted mortality (Jain et al., 2009). *P. aeruginosa* is especially vulnerable to genetic alterations resulting antibiotic resistance and consequent difficulties in immunocompromised individuals. The capacity of *P. aeruginosa* to utilize high levels of inherent and acquired resistance mechanisms to resist most antibiotics has made eradication increasingly challenging (Salomoni et al., 2017). Hence, there is an increasing demand for the development of novel alternatives to combat MDR pathogens (Khalil et al., 2015a, 2020). Under this scope, several metallic nanomaterials have superior antibacterial activity against MDR bacteria over traditional antibiotics. As far as it is aware, many studies have reported nanotechnology, and the use of some metal nanomaterials in medicine are gaining more attention as new, effective alternatives to commercial antibiotics, particularly against MDR isolates (Deng et al., 2016; El Din et al., 2016; Thapa et al., 2017). Nanoparticle-antibiotic combinations have many benefits, such as reducing the dosage amount of both agents, which may minimize toxicity and increase the antimicrobial properties (Buszewski et al., 2018). Therefore, such a combination may increase antibiotic concentrations at the place of antibiotic-microbe contact promoting increased affectivity (Rai et al., 2012; Lozovskis et al., 2020). The nanoparticles of the certain metal display different unique properties compared to that of their large-size counterparts (Gold et al., 2018). Nanomaterials are a distinct category of materials with unique properties and a wide range of applications in a different field. Their increased specific surface area enables interaction with bio-organic components on the viable cell surface of microorganisms, resulting in structural denaturation in the microbial cells (Alavi and Rai, 2019).

Metal nanoparticles have been synthesized using a number of methods, including biological processes, chemical reduction of metal salts using reductive chemicals, sonochemical deposition, photochemical reduction, microwave irradiation, and laser irradiation (Singh et al., 2015; Ali et al., 2017; Lozovskis et al., 2020). Biosynthesis of nanoparticles is an emerging highlight of the convergence of nanotechnology and biotechnology, which has gained rising attention due to the growing need to improve eco-friendly materials synthesis technologies (Kalishwaralal et al., 2010; Deepak et al., 2011; Husseiny et al., 2015). At the same time, several researchers (El-Shanshoury et al., 2012; Saravanan et al., 2017) have documented the usage of biosynthesis of nanoparticle metals by microorganisms as an alternative way to substitute traditional chemical processes to provide eco-friendly, low-cost, small-scale production methods. Others have reported a range of pitfalls that would minimize the potential to use microorganisms such as bacteria and fungi to synthesis of metals nanoparticles on a large scale (Kalimuthu et al., 2008). Binding the synthesized nanoparticles to the producing organism’s cell membranes often complicates the process of purifying these biomass nanoparticles, which requires critical measures to break down biomass. On the other side, the technique of chemical reduction utilizing organic or inorganic reduction agents for the chemical synthesis of nanoparticles is commonly used due to its advantages in the manufacture of low-cost non-aggregated nanoparticles and the potential to be used in large-scale production (Khan et al., 2019; Bezza et al., 2020).

Several experiments have reported the *in vitro* antimicrobial activity of silver nanoparticles (Ag-NPs). The use of silver ions has long been known in the treatment of skin infections (Rai et al., 2009; Oryan et al., 2018; Wasef et al., 2020). To the best of our knowledge, there have been no reports, to date, on the effectiveness of *in vivo* Ag-NPs in combination with neomycin as a novel antimicrobial candidate in the treatment of MDR *P. aeruginosa*-infected burn wounds. The results of this study thus open up a new viewpoint on the *in vivo* and *in vitro* antibacterial activities of antibiotic combined metallic nanomaterials that can selectively fight MDR pathogens induced by burn wound infections without impacting healthy mammalian cells.

## Materials and Methods

### Isolation and Identification of Clinical Isolates

**FIGURE 1:**
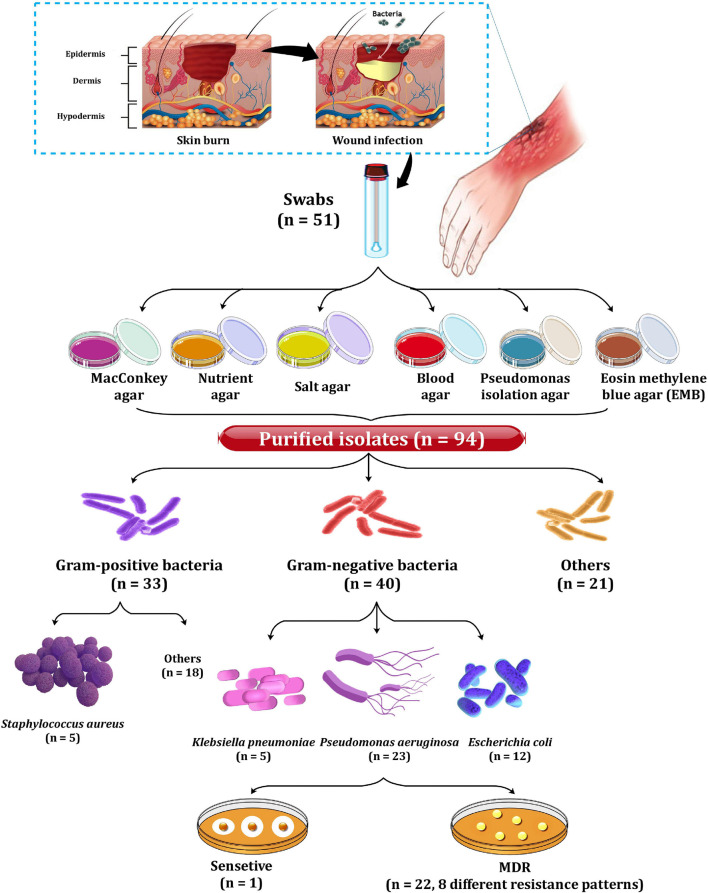
Incidence of the recovered bacterial isolates.

As depicted in Figure 1, a total of 51 swabs were collected from patients with burn wound infection (2nd and 3rd degree of burn) in various hospitals, including the Burn Unit of Tanta Emergency Hospital, Mubarak Hospital, and El-Salam Hospital in Tanta, Egypt (Khalil et al., 2017). The study and consent form have been approved by the Tanta University Hospital’s Research Ethical Committee Centers. All swabs were cultured immediately after collection on nutrition agar, MacConkey agar, mannitol salt agar, eosin methylene blue agar, and blood agar plates. Purification of the growing bacteria was then achieved *via* sub-culturing. As a result, 94 bacterial isolates were recovered and further subjected to biochemical tests for identification. The selected Gram-negative bacterial isolates were sub-cultured on MacConkey agar. The resulting non-lactose fermenting isolates were further sub-cultivated on cetrimide agar plates to preliminary collect of *Pseudomonas aeruginosa* (Figure 1). The obtained isolates were identified following Holt et al. (1994). For further confirmation of isolates’ identity, API 20E identification was also performed.

### Determination of the Antimicrobial Susceptibility of the Tested Isolates

*Pseudomonas aeruginosa* isolates (*n* = 23) were tested for their susceptibility to nine different antibiotics using disk diffusion technique. Carbenicillin (Py), piperacillin (PRL), cefepime (FEP), aztreonam (ATM), imipenem (IPM), gentamicin (GEN), neomycin (N), levofloxacin (LEV), and tetracycline (TE) were the antibiotics examined. The plates were incubated for 18 h at 37°C. The sensitivity was evaluated after incubation by measuring the zone of growth inhibition (mm). The results were interpreted as sensitive (S), intermediate (I), or resistant (R) based on the European Committee on antimicrobial susceptibility testing (EUCAST) (2012) clinical breakpoint. The term multidrug-resistant (MDR) applies to a bacterium that acquired non-susceptibility to at least one agent in three or more antimicrobial categories (Magiorakos et al., 2012), while pandrug-resistance (PDR) was identified as resistance to all agents in all antimicrobial classes tested (Ali et al., 2021).

### The Antibacterial Efficacy of the Synthesized Nanoparticles Alone or in Combination With Antibiotics Against *Pseudomonas aeruginosa*

Ag-NPs were synthesized by the chemical reduction and the precipitation processes, respectively, and were characterized as previously stated by Khalil et al. (2017). The antibacterial activity of Ag-NPs or antibiotics alone was compared to that of their Ag-NPs combinations against bacterial isolates using the disk diffusion method previously described (Khalil et al., 2017). Standard antibiotic disks impregnated with Ag-NPs (1.7 μg/ml) were used. Plates were properly labeled and incubated at 37°C for 18 h. After incubation, the diameters of the inhibitory zones (mm) around the disks were determined. All experiments have been performed in triplicates. The fold increase in the diameter of each antibiotic’s inhibitory zone following combination with Ag-NPs was calculated using Equation 1, as shown below:


(1) $$\mathrm{The}\mathrm{fold}\mathrm{increase}=\left(b^{2}-a^{2}\right)/a^{2}$$

Where; *a*: is the inhibition zone of antibiotic alone, while *b*: is the inhibition zone of antibiotic plus nanoparticles (Sindhu et al., 2013).

### Determination of Minimum Inhibitory Concentrations

The minimum inhibitory concentrations (MICs) values of Ag-NPs alone, neomycin, and its combination with Ag-NPs against two selected *P. aeruginosa* isolates (P_8_ and P_14_) were calculated using resazurin-based microtiter dilution assay (Wiegand et al., 2008; Abdelaziz et al., 2014).

#### Determination of Ag-NPs Stock Concentration

Firstly, the stock Ag-NPs colloidal suspension was prepared by the chemical reduction of silver nitrate solution in Deionized Distilled Water (DDW) as previously described by Khalil et al. (2017). The concentration of the prepared stock Ag-NPs colloidal suspension was determined by Equation 2 as described by Liu et al. (2007).


(2) $$\text{C}=\frac{n_{t}}{n.v.n_{a}}$$

Where;
C: molar concentration of nanoparticle solution,
*n*_*t*_: total number of silver atoms added as AgNO_3_= 1 M,
*n*: number of atoms per nanoparticle (calculated by Equation 3),
*v*: volume of the reaction solution in liter,
*n*_*a*_: Avogadro’s number (=6.023 × 10^23^).

(3) $$\text{N}=\frac{\pi \rho D^{3}}{6M}n_{a}$$

Where;
N: number of atoms per nanoparticles,
π = 3.14,
ρ: density of face-centered, cubic (fcc) silver (=10.5 g/cm^3^),
D: average diameter of nanoparticles (measured by TEM),
M: atomic mass of silver (=107.868 g),
*n*_*a*_: Avogadro’s number (=6.023 × 10^23^).

#### MIC of Ag-NPs

The previously prepared stock colloidal solution of Ag-NPs (1.7 μg/ml) was diluted with sterile dist. H_2_O to obtain the working solutions, each having a concentration two times the appropriate final concentrations obtained by diluting the samples with MHB media in the microtiter plates. The 96-well microtiter plates were loaded with various concentrations Ag-NPs (100 μl each) and bacterial inoculum (100 μl, 1 × 10^5^ CFU/ml). Plates were then incubated at 37°C for 18 h (Chan and Mashitah, 2013). After incubation, 20 μl of membrane-filtered resazurin dye (0.1% w/v in dist. H_2_O) was applied to each well, and the plates were incubated in an incubator shaker (37°C, 1 h). The transformation of purple resazurin into a fluorescent pink to red showed actively metabolizing cells, while the presence of dark blue suggested full suppression of bacterial growth in microtiter plate wells. The MICs were detected as the lowest concentrations of Ag-NPs in the wells remained blue. All experiments were conducted in triplicates (Lipuma et al., 2009; Singh et al., 2013; Abdelaziz et al., 2014).

#### MIC of Neomycin Alone or Combined With Ag-NPs Against the Tested Isolates

Neomycin (aminoglycosides) was chosen for the following *in vivo* study, as neomycin is commonly used in the topical treatment of many skin infections, including burns. The MIC value of the antibiotic was determined either individually or combined with Ag-NPs against *P. aeruginosa* isolates (P_8_ and P_14_). Antibiotic dry powder (10.000 μg/ml) was mixed in DDW to prepare a stock of the antibiotics, which was subsequently sterilized using bacterial filters (pore size, 0.22 μm). To prepare twofold serial dilutions of antibiotics, the stock concentrations were either diluted with sterile dist. H_2_O for detecting MIC of free drug or diluted with Ag-NPs suspension (at sub-inhibiting concentration) for the combination. Every working solution had a concentration two times the final concentrations required, which was achieved by diluting the working samples with MHB media. The 96-well microtiter plates were loaded with 100 μl of each antibiotic concentration, either alone or in combination with Ag-NPs, and inoculated with 100 μl of bacterial culture (10^5^ CFU/ml). The plates were incubated at 37°C for 18 h. MICs were observed by broth microdilution technique using resazurin dye as stated above.

### *In vivo* Evaluation of the Efficacy of Ag-NPs–Antibiotics Combination in the Treatment of Induced Bacterially Infected Burns

The effectiveness of Ag-NPs alone and in combination with the selected antibiotic (neomycin) in encouraging the healing of induced *P. aeruginosa*-infected burn-in experimental animal models (Swiss albino mice) was studied and compared to that of the antibiotic alone.

#### Experimental Animals

Eight weeks old, eighty male Swiss albino mice weighing 18–24 g were employed in this study. The research was performed in compliance with the ethical guidelines approved by the Animal Ethics Committee Guide Lines of Tanta University. The mice had free access to food and water on a rodent diet. They were housed in individual special cages and kept under a 12 h light-dark period at 22 ± 2°C in the animal house of the Faculty of Science, Tanta University.

#### Preparation of Different Drug Formulations

The gel formulations used in the *in vivo* experiment were formulated following Purushothamrao et al. (2010), as given in Supplementary Table 1. Briefly, methylcellulose (4.5 g) was spread in 50% of the liquid medium (sterile dist. H_2_O or colloidal Ag-NPs) retained at 80°C with intense mixing to achieve homogenous polymer suspension. The remainder of the cold medium was applied to the received polymer by continuous mixing away from heat until the desired homogeneity was achieved. For antibiotic-containing formulations, the dried powder of neomycin was dissolved in the cold portion of the liquid medium before being applied to the polymer. All prepared gel formulas were held overnight at 4°C to ensure full polymer swelling. However, the liquid spray formula was prepared by dissolving neomycin directly in the colloidal Ag-NPs.

#### Animal Model of Burn Infection

To evaluate the effectiveness of various drug formulations, mice models were classified into seven groups (G1-G7), including ten mice in each group. G1 (negative control) comprised neither burned nor infected mice. In G2 and G3 (positive controls), mice were burnt and get infected without receiving any treatment (G2) or treated with Placebo gel only (G3). The mice of G4 were burnt, infected, and treated with Ag-NPs gel, while the mice of G5 were burnt, infected, and treated with neomycin gel. G6 and G7 (neomycin-Ag-NPs gel and spray formulas, respectively) in which mice were burnt, microbial suspensions were applied, and the mice received treatment with neomycin-Ag-NPs gel (G6) and neomycin-Ag-NPs spray (G7), respectively. On the other hand, topical toxicity of the drug formulations used in this study (Ag-NPs gel, neomycin gel, neomycin-Ag-NPs gel, neomycin-Ag-NPs spray, and Placebo gel base) was performed following the method described by Basketter et al. (2004). Drug formulations were administered directly to shaved skin once daily for 1 week before the experiment. They were observed to ensure that the components used were not skin irritants even under exaggerated exposure conditions.

#### Induction of Burn Injuries and Bacterial Infection in Mice

The day before the formation of burns, mice are shaved on the back. The next day, non-lethal full-thickness third-degree burns were produced on their backs by adding two preheated (92–95°C) brass blocks to the opposite sides of the elevated skin fold on the back of the mouse for 60 s. The combined brass block area was 20 mm by 10 mm, with an area of 200 mm^2^, equivalent to 5% of the total body surface area (TBSA) (Huang et al., 2011). Mice were resuscitated intraperitoneally with 0.5 ml sterile saline solution immediately after developing burns in the groups examined (G2-G7). Burns was permitted to cool down for 5 min, and then 40 μl of freshly prepared *P. aeruginosa* (P_8_) (10^8^ CFU/ml) was added to the surface of each burn using a micropipette. After 2 h of infecting the burning regions, various formulations were added to the infected areas (initial treatment time). All the treatments were performed once daily for 10 days (end period of treatment). Animals were tracked throughout the treatment and post-treatment periods until the 24th day (end time of the experiment) to measure the healing improvement over time. The mortality rate, the bacterial count in the skin and blood, the percentage of wound contraction, and the histopathological analysis of the lesions by light microscope and transmission electron microscope (TEM) were performed for determining the *in vivo* efficacy of the various drug formulations tested following the previous methods (Sundaramoorthi et al., 2011; Abdelaziz et al., 2014).

#### Bacterial Count in Skin and Blood Samples

The bacterial count in the skin of the various groups of infected mice relative to the control groups was conducted by taking a swab from each mouse at the initial and end period of treatment. On the surface of the ceramide agar medium, the swabs were cultivated. In a bacterial count, blood samples from mice were collected through eye-bleeding at the end of treatment (Forbes et al., 2010). Plates were incubated at 37°C for 18 h, and the number of colonies was determined based on the Equation 4. All the tests were performed in triplicates.


(4) $$\mathrm{Number}\mathrm{of}\mathrm{bacteria}\left(\mathrm{CFU}/\mathrm{ml}\right)=\frac{\mathrm{average}\mathrm{number}\mathrm{of}\mathrm{bacterial}\mathrm{colonies}}{\mathrm{amount}\mathrm{plated}}\times \mathrm{dilution}\mathrm{factor}$$

#### Measuring of Wound Contraction Percentage

Measurement of the wound region (till day 24 of post-treatment) was expressed as a unit (mm^2^). In contrast, the wound contraction was stated as the percentage reduction of the initial wound size, determined from the following equation (Sundaramoorthi et al., 2011).


(5) Woundcontraction%=Woundareaday 0-WoundareadaynWoundareaday 0×100

#### Histological Examination of Skin Samples

Skin samples of mice in all groups studied were examined using a light microscope and a TEM to detect the influence of Ag-NPs alone and combined with neomycin in various formulations on the skin layers’ morphology, arrangement, and ultra-structures. Photography was carried out using TEM (JEOL, JEM-100 SX, Japan) in the Electron Microscope Unit, Faculty of Medicine, Tanta University.

#### Examination of the Bacterial Cells Ultra-Structures

Cells of the selected *P. aeruginosa* isolate (P_8_) were analyzed by TEM after treatment with Ag-NPs alone, neomycin alone, and neomycin-Ag-NPs combination for 12 h and compared to untreated cells. The concentrations of Ag-NPs and antibiotics used in this experiment were adjusted to their sub-inhibitory concentrations as determined by their MICs. In each culture, the bacterial inoculum was set to 10^8^ CFU/ml.

### Statistical Analysis

All experiments were performed in triplicates. The results were analyzed by NCSS 2020 (NCSS, LIC, UT, United States) and Minitab software version 19.2020.1 (Minitab Inc., United States). To evaluate statistical significance, ANOVA with Tukey–Kramer multiple comparisons and *t*-Student’s tests were applied at *P*-value ≤ 0.05.

## Results

**FIGURE 2:**
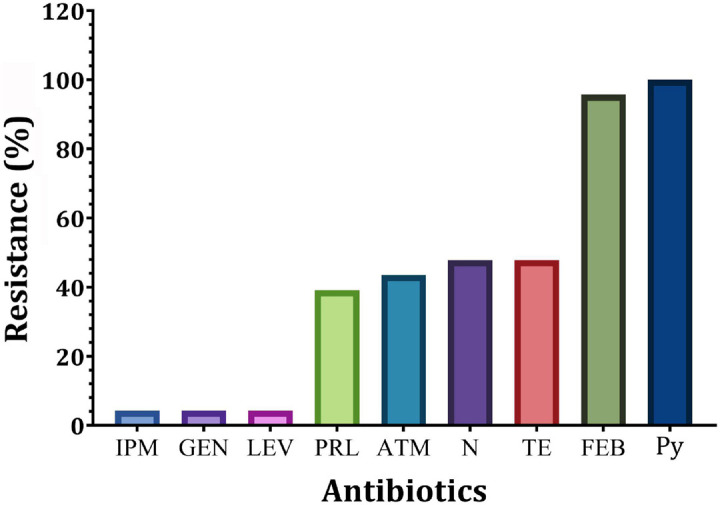
Prevalence of resistance of *P. aeruginosa* isolates to different antibiotics. IPM, imipenem; GEN, gentamicin; LEV, levofloxacin; PRL, piperacillin; N, neomycin; TE, tetracycline; ATM, aztreonam; FEP, cefepime; Py, carbenicillin.

Out of 94 bacterial isolates screened from 51 clinical swab samples taken from human burn wound infection, a total of 40 Gram-negative bacterial isolates were recovered (Figure 1). Based on biochemical and API 20E identification, these isolates included 23 *P. aeruginosa*, 12 *E. coli*, and 5 *K. pneumoniae* isolates. Only *P. aeruginosa* isolates were selected for further study. The majority of *P. aeruginosa* isolates were found to be highly resistant to Py (100%), followed by FEB (95.7%), while the lowest resistance (4.3%) was observed against IPM, GEN, and LEV (Figure 2). According to susceptibility testing to nine different antibiotics showed that all *P. aeruginosa* (95.7%) isolates tested except for P_19_ isolate were MDR (resistant to at least one agent in three or more antimicrobial categories) (Table 1). As depicted in Table 1, five resistance patterns (I-V) have been obtained. The pattern I was categorized into three classes (Ia, Ib, and Ic) with different antibiotics. Of these patterns, three isolated were selected randomly; one from each class. As a result, the isolates P_1_ (Py, FEB, and TE), P2 (Py, FEB, and LEV), and P_11_ (Py, FEB, and N) were selected to represent pattern Ia, pattern Ib, and pattern Ic, respectively (Table 1). Similarly, *P. aeruginosa* isolate P_10_ (Py, FEB, PRL, and, ATM) was selected randomly to represent pattern IIa, while P_8_ (Py, FEB, N, and TE) represents the resistance pattern IIb (Table 1). On the other hand, five different antibiotics represented pattern III with two *P. aeruginosa* isolates (P_6_ and P_18_). Of these isolates, P_18_ with the resistance pattern (Py, FEB, PRL, ATM, and TE) was selected. However, among three *P. aeruginosa* isolates that represent the resistance pattern IV with six different antibiotics, isolate P_14_ (Py, FEB, PRL, ATM, N, and TE) was randomly selected. Interestingly, *P. aeruginosa* isolate P_16_ showed resistance to the all antibiotics tested (Py, FEB, PRL, ATM, IPM, GEN, N, LEV, and TE), representing the only isolate in the resistance pattern V. As resistance to all tested antibiotics is defined as PDR. Hence, *P. aeruginosa* P_16_ is PDR isolate, while the other isolates P_1_, P_2_, P_11_, P_10_, P_8_, P_18_, and P_14_ are MDR *P. aeruginosa* (Table 1).

**TABLE 1:** The antimicrobial resistance patterns of MDR *P. aeruginosa* isolates.

Pattern code		Resistance pattern profile*	Number of MDR isolates (*n* = 22)	Isolate code exhibited by pattern
I	a	Py, FEB, TE	3	P1, P15, P20
	b	Py, FEB, LEV	2	P12, P17
	c	Py, FEB, N	3	P2, P23,P11
II	a	Py, FEB, PRL, ATM	3	P10, P5, P3
	b	Py, FEB, N, TE	5	P8, P7, P9, P21, P22
III		Py, FEB, PRL, ATM, TE	2	P18, P6
IV		Py, FEB, PRL, ATM, N, TE	3	P14, P13, P4
V		Py, FEB, PRL, ATM, IPM, GEN, N, LEV, TE	1	P16

*IPM, imipenem; GEN, gentamicin; LEV, levofloxacin; PRL, piperacillin; N, neomycin; TE, tetracycline; ATM, aztreonam; FEP, cefepime; Py, carbenicillin. MDR, multidrug resistant.

**FIGURE 3:**
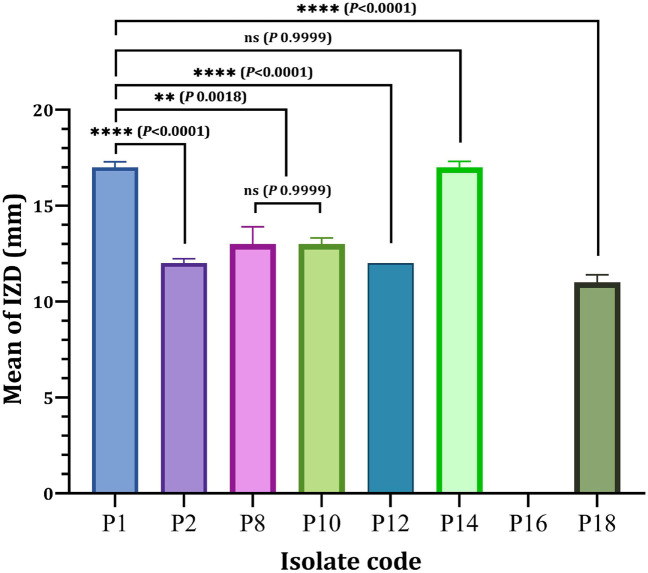
Antibacterial activity of Ag-NPs against selected *P. aeruginosa* isolates. IZD, inhibition zone diameter (mm); ns, non-significant. **P < 0.01, ****P < 0.0001 and *P*-value ≤ 0.05 is significant.

Eight *P. aeruginosa* isolates (P_1_, P_2_, P_8_, P_10_, P_12_, P_14_, P_16_, and P_18_) were randomly chosen from the eight different resistance patterns, one isolated from each pattern *in vitro* antibacterial effectiveness of nanoparticles or different antibiotics-nanoparticles combinations against clinical isolates. As depicted in Figure 3, a significant difference in the antibacterial efficacy of Ag-NPs relative to the selected eight isolates was obtained since only P_16_ showed resistance to Ag-NPs compared to other isolated tested. The antibacterial activity of Ag-NPs against tested isolates showed different inhibition zone diameters (IZD), ranged from 11 to 17 mm. P_1_ and P_14_ isolates achieved the maximum IDZ values when compared with other isolated. However, no significant difference (*P* < 0.9999) was observed in the IZD of P1 and P_14_ (Figure 3). On the other hand, P_1_ (17 ± 0.29 mm) showed a significant higher IZD (*P* < 0.0001) as compared to P_2_ (12 ± 0.23 mm), P_12_ (12 ± 0.00 mm) and P_18_ (11 ± 0.40 mm). In contrast, MDRP8 and MDRP10 showed no significance in terms of IZD (*P* < 0.9999) (Figure 3).

**FIGURE 4:**
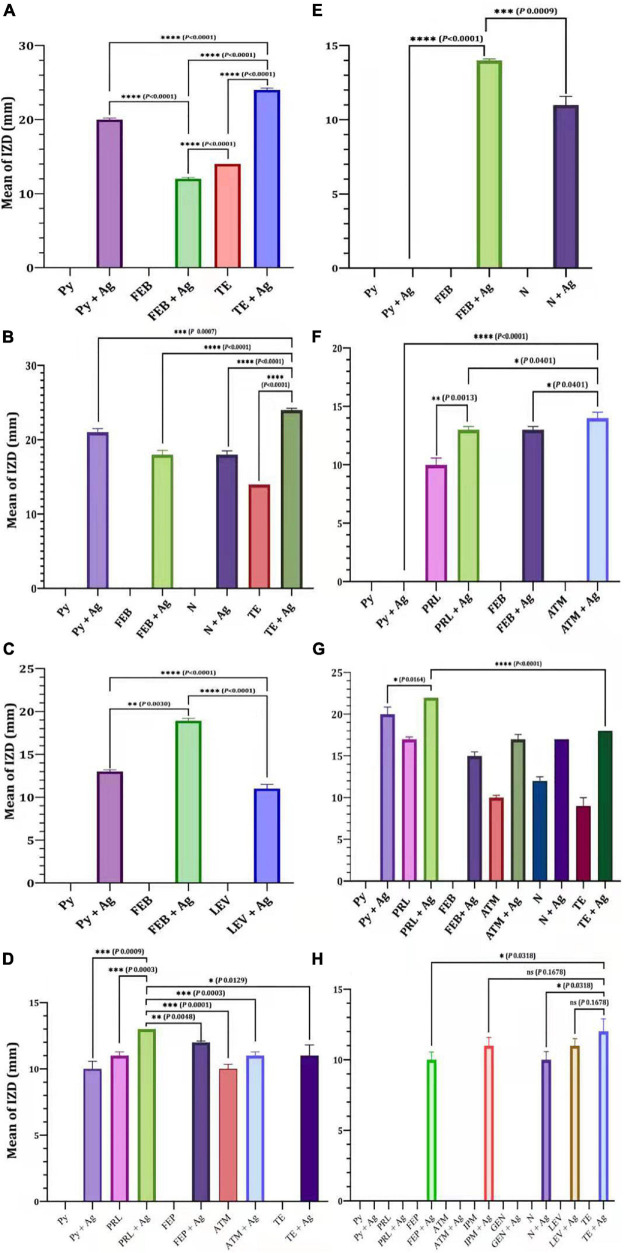
Efficacy of antibiotics alone or in combination with Ag-NPs against selected MDR *P. aeruginosa* isolates. *P. aeruginosa* P_1_ **(A)**, *P. aeruginosa* P_8_ **(B)**, *P. aeruginosa* P_12_ **(C)**, *P. aeruginosa* P_18_ **(D)**, *P. aeruginosa* P_2_ **(E)**, *P. aeruginosa* P_10_ **(F)**, *P. aeruginosa* P_14_ **(G)**, *P. aeruginosa* P_16_ **(H)**. IPM, imipenem; GEN, gentamicin; LEV, levofloxacin; PRL, piperacillin; N, neomycin; TE, tetracycline; ATM, aztreonam; FEP, cefepime; Py, carbenicillin; ns, non-significant. *P*-value ≤ 0.05 is significant.

The efficacy of antibiotics alone or in combination with Ag-NPs against selected MDR *P. aeruginosa* isolates was evaluated (Figure 4). For the isolate MDRP1, the mean of IZD of Py combined with Ag-NPs was significantly higher than that of FEB combined with Ag-NPs (*P* < 0.0001) (Figure 4A). On the other hand, the mean of IZD of TE combined with Ag-NPs was significantly higher than that of Py + Ag-NPs (*P* < 0.0007), FEB + Ag-NPs (*P* < 0.0001), N + Ag-NPs (*P* < 0.0001), and TE only (*P* < 0.0001) (Figure 4B). FEB combined with Ag-NPs achieved a significantly higher IZD mean (*P* < 0.0001) when compared with Py combined with Ag-NPs for *P. aeruginosa* P_12_ (Figure 4C). In the case of *P. aeruginosa* P_16_, the mean of IZD achieved by PRL combined with Ag-NPs was significantly higher than those of FEP combined with Ag-NPs (*P* < 0.0048), ATM (*P* < 0.0001), ATM combined with Ag-NPs (*P* < 0.0003), and TE combined with Ag-NPs (*P* < 0.0129), PRL (*P* < 0.0003) as well as Py combined with Ag-NPs (*P* < 0.0009) as shown in Figure 4D. A significantly higher IZD mean was observed for *P. aeruginosa* P2 by FEP combined with Ag-NPs compared to N combined with Ag-NPs (*P* < 0.0009) (Figure 4E). On the other hand, the IZD mean values of ATM combined with Ag-NPs and PRL combined with Ag-NPs were significantly higher than that of FEP combined with Ag-NPs (*P* < 0.0401) and PRL (*P* < 0.0013), respectively, as achieved against *P. aeruginosa* P_10_ (Figure 4F). Similarly, PRL combined with Ag-NPs revealed a significantly higher IZD mean when compared with Py combined with Ag-NPs (*P* < 0.0164) or TE combined with Ag-NPs (*P* < 0.0001), as observed in the case of *P. aeruginosa* P_14_ (Figure 4G). For *P. aeruginosa* P_18_, TE combined with Ag-NPs achieved a significantly higher IZD mean over N combined with Ag-NPs (*P* < 0.0318) and FEP combined with Ag-NPs (*P* < 0.0318). On contrary, no significant difference (*P* < 0.1678) in the mean of IZD was observed between TE combined with Ag-NPs and LEV combined with Ag-NPs or IPM combined with Ag-NPs (Figure 4F).

**FIGURE 5:**
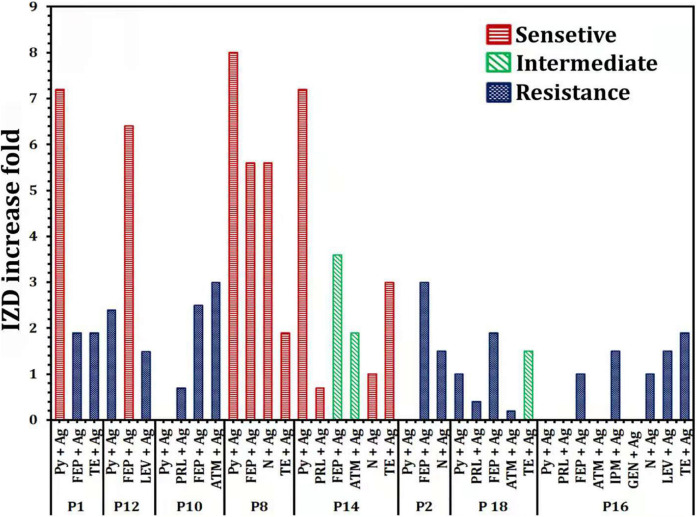
Increase fold of antibacterial activity of antibiotics alone or in combination with Ag-NPs against selected *P. aeruginosa* isolates. IPM, imipenem; GEN, gentamicin; LEV, levofloxacin; PRL, piperacillin; N, neomycin; TE, tetracycline; ATM, aztreonam; FEP, cefepime; Py, carbenicillin.

Increase fold of antibacterial activity of antibiotics alone or in combination with Ag-NPs against selected *P. aeruginosa* isolates were also determined (Figure 5). P_16_ revealed an improvement in the sensitivity of antibiotic-nanoparticle combination with gating IZD increased from 0.0 mm (with Py and FEB) to 10 ± 0.58 and 12 ± 0.00 mm (with antibiotic-Ag-NPs combination) (Figure 4), with fold increase 1 and 1.9, respectively, but still within the resistance susceptibility profile (Figure 5). The assessment of the antibacterial efficacy of nanoparticle-antibiotic combinations against selected *P. aeruginosa* isolates indicated that the highest synergism expressed as increasing susceptibility to isolate was recorded with P_8_ and P_14_ isolates (Figure 5). In general, the data obtained indicated a substantial improvement in the susceptibility of P_8_ to become sensitive to Py, FEP, N, and TE, with the highest fold increase in the inhibition zone of 8 (Figure 5). Regarding P14 that showed the antibiotic resistance pattern (Py, PRL, FEP, ATM, N, TE), it has become significantly susceptible to tested antibiotics with their combination with Ag-NPs; Py combined with Ag-NPs (20 ± 0.87 mm, *P* < 0.0001), PRL combined with Ag-NPs (22 ± 0.00 mm, *P* < 0.0001), N combined with Ag-NPs (17 ± 0.00 mm, *P* < 0.0001), TE combined Ag-NPs (18 ± 0.00 mm, *P* < 0.0001) that they were initially resistant (Figures 4, 5). In addition, the P_1_ susceptibility changed from Py resistant to sensitive one with a fold increase of 7.2, and the highest increase in susceptibility of 6.4 was reported for P_12_ with FEB combined with Ag-NPs (Figure 5).

**FIGURE 6:**
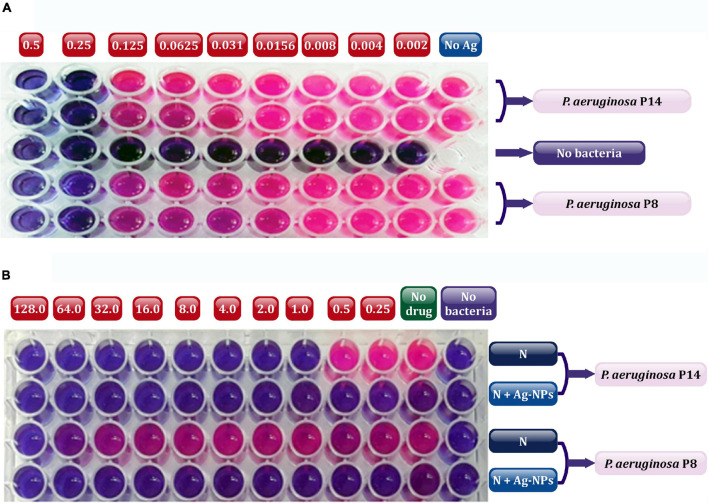
Determination of MIC values using resazurin microtiter plates showing Ag-NPs **(A)**, and neomycin alone or in combination with Ag-NPs **(B)** against selected P8, and P14 isolates. In the plate **(A)**, the numbers above the plate indicate the concentration of Ag-NPs (μg/ml) applied to each wells column; no Ag, without addition of Ag-NPs (positive control). In panel **(B),** the numbers above the plate indicate the concentration of neomycin (μg/ml) added to each column of wells; no drug, without addition of neomycin (positive control); no bacteria, without bacterial addition (negative control). N, neomycin; Ag-NPs, silver nanoparticles. The red color indicated actively metabolizing cells and dark blue color indicated the complete inhibition of bacterial growth in the wells of microtiter plates.

The *in vivo* efficacy of Ag-NPs alone and combined with a selected topical antibiotic (neomycin) encourage healing in experimental animals of induced burn infected with the selected bacterial isolates MDRP8 and MDRP14. The colloidal stock solution of Ag-NPs was prepared, and the measured concentration was found to be 1.7 μg/ml. Based on the data obtained from the MICs of Ag-NPs (Figure 6), both isolates were found to be extremely susceptible to Ag-NPs even at very low concentrations with a MIC value of 0.25 μg/ml (Figure 6A). Neomycin MICs were calculated independently or in combination with Ag-NPs against the selected two *P. aeruginosa* isolates using resazurin-based microtiter dilution assay (Figure 6B). The finding showed that neomycin MIC was 128 μg/ml and 1 μg/ml, respectively, against P_8_ and P_14_. These values decreased to ≤0.25 μg/ml by the addition of sub-inhibitory concentration of Ag-NPs. Generally, the MICs for P_14_ (1, ≤0. 25 μg/ml), and for P_8_ (≥128, ≤0.25 μg/ml) were for neomycin.

Neomycin was used for *in vivo* experiments in different formulations of drugs to treat burns induced by MDR *P. aeruginosa* P_8_ in experimental animals. Such formulations were added directly to unburned mice skin. No symptoms of toxicity were detected using the drug preparations under investigation (data not shown). As depicted in Table 2, the mice G7 treated with neomycin-Ag-NPs spray exhibited the greatest antibacterial activity as seen by the highest reduction in the level of bacterial at the initial period of treatment compared to the other groups with full bacterial growth inhibition on the end time of treatment. However, the lowest antibacterial activity was observed in G3 (treated with placebo base gel) followed by G5 (treated with neomycin gel). The antibacterial activity of G4 (treated with Ag-NPs gel) was relatively higher than that of G5 (treated with neomycin gel), yielding a bacterial count of 2.6 × 10^3^ and 4.1 × 10^3^ CFU/ml, respectively, at the end of treatment (Table 2). As far as blood samples were concerned, there was no evidence of the bacterial presence in the blood at the end time of infection in all groups or the untreated group. It was also noticed that the highest percentage of mortality was observed in G2 (positive control; mice were burnt and get infected without receiving any treatment), since 30% of mice were reported, followed by G5 (treated with neomycin gel) and G3 (positive control; mice were burnt, get infected and received treatment with placebo gel only) with a mortality percentage of 10% per mice (Table 2).

**TABLE 2:** Bacterial skin count and mortality of various groups examined under different treatment conditions.

Mice groups	^#^Mean bacterial count (CFU/ml)		Mortality (%)
	1st stage	2nd stage	
G1	0.0 ± 0.0^a^	0.0 ± 0.0^a^	0.0 ± 0.0^a^
G2	2.75 × 10^4^ ± 0.5^b^	1.4 × 10^4^ ± 0.5^b^	29.6 ± 0.5^c^
G3	2.58 × 10^4^ ± 0.5^b^	1.18 × 10^4^ ± 0.5^b^	9.6 ± 0.5^b^
G4	5.2 × 10^3^ ± 0.8^e^	2.6 × 10^3^ ± 0.6^c^	0.0 ± 0.0^a^
G5	6.2 × 10^3^ ± 0.4^f^	4.1 × 10^3^ ± 0.8^e^	9.6 ± 0.5^b^
G6	4.9 × 10^3^ ± 0.6^e^	0.15 × 10^3^ ± 0.4^a^	0.0 ± 0.0^a^
G7	3.8 × 10^3^ ± 0.8^c^	0.0 ± 0.0^a^	0.0 ± 0.0^a^
F-value	41.545***	30.271***	2.53***

Independent *t*-test and values are mean of three replicates ± SD; 1st stage, after 3 days of initial treatment; 2nd stage, at the treatment end time. Means in the same column followed by different letters significantly different at *P*<0.05, ***P < 0.001.

*^#^Number of bacteria (CFU/ml) = average number of bacterial colonies/amount plated × dil. Factor.*

*G1 (negative control) comprised neither burned nor infected mice.*

*G2 and G3 (positive controls), mice were burnt, and get infected without receiving any treatment (G2) or treated with Placebo gel only (G3).*

*The mice of G4 were burnt, infected, and treated with Ag-NPs gel, while the mice of G5 were burnt, infected, and treated with neomycin gel. G6 and G7 (neomycin-Ag-NPs gel and spray formulas, respectively) in which mice were burnt, microbial suspensions were applied, and the mice received treatment with neomycin-Ag-NPs gel (G6), and neomycin-Ag-NPs spray (G7), respectively.*

**FIGURE 7:**
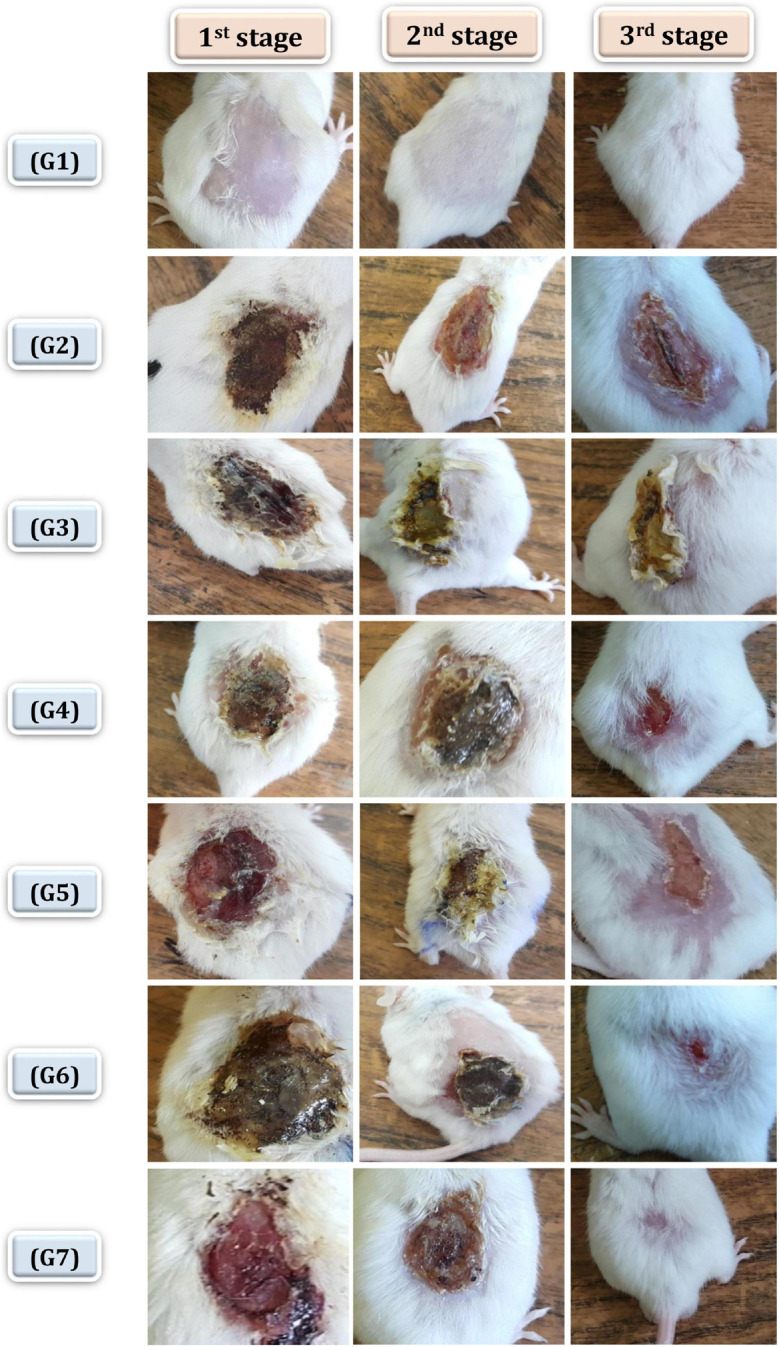
Healing progress in mice groups after different days of treatment. G1 normal group (negative control), G2 infected and untreated (positive control), G3 infected, and treated with placebo base gel (positive control), G4 infected, and treated with Ag-NPs gel, G5 infected, and treated with neomycin gel, G6 infected, and treated with neomycin-Ag-NPs gel, G7 infected, and treated with neomycin- Ag-NPs spray. 1st stage: after 3 days of initial treatment, 2nd stage, at the treatment end time, 3rd stage: at the end of experiment.

**FIGURE 8:**
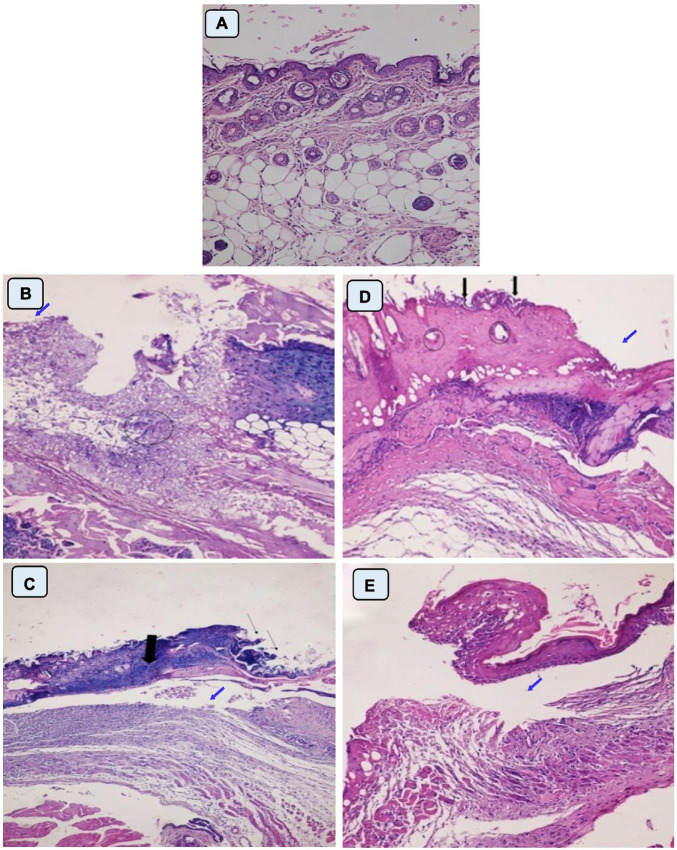
Histopathological examination of skin mice of control groups during 1st stage **(B,C)** and 2nd stage of treatment **(D,E)**. G1 is negative control comprised neither burned nor infected mice **(A)**. G2 is positive control; mice skin were burnt and get infected with *P. aeruginosa* P8 without receiving any treatment **(B,D)**. G3 is positive control; mice skin were burnt, and get infected with *P. aeruginosa* P8 and received treatment with placebo gel only **(C,E)**. Magnification power (200×). **(B)** Loss of epidermis (blue arrow), distortion in the dermis layer with irregular distribution of collagen (circle). **(C)** Epidermis loss (thin arrow), underlying massive infiltration (thick arrow), and edema space in the dermis layer (blue arrow). **(D)** Irregular epidermis layer, infiltration with inflammatory cells (black arrow) with distortion in the glands within the dermis layer (circle), and large lytic area (blue arrow). **(E)** Separation of epidermis from the dermis layer with underlying inflammatory cells and irregular distribution of collagen in dermis layer underlying edema space (blue arrow).

In this study, healing progress in mice groups after different days of treatment was also determined (Figure 7). Neomycin-Ag-NPs spray (G7) demonstrated the highest healing activity as seen by the maximum percentage of wound contraction (96.7% of the wound area contracted) at the end of the experiment, followed by neomycin-Ag-NPs gel (G6), where 88.8% of the wound area was contracted. In comparison, the healing efficacy of Ag-NPs gel in G4 was greater than that of neomycin gel in G5 with a wound contraction percentage of 82.5 and 71.4%, compared with 59.9% for G2 (positive control; mice were burnt and get infected without receiving any treatment) and 61.3% for G3 (positive control; mice were burnt, get infected and received treatment with placebo gel only). On the other hand, the investigation of normal skin sections of G1 (negative control) displays normal epidermis and dermis layers where the collagen was normally distributed within the dermal layer, with the inclusion of normal secretions (Figure 8A). In the 1st stage of treatment, the skin of G2 (positive control; mice were burnt and get infected without receiving any treatment) seemed to be seriously damaged with complete loss of the epidermal layer, while the underlying dermal layer appeared to be distorted by rarified collagen and infiltrated by inflammatory cells (Figure 8B). In addition, G3 (positive control; mice were burnt, get infected and, received treatment with Placebo gel only) showed no significant difference when compared with G2 since the epidermal layer was completely lost, and the dermal layer was severely harmed (Figure 8C). However, in the 2nd stage (at the end of the treatment), the skin was severely damaged in G2 and G3, the epidermis, as well as dermis layers, were distinctly distorted, separated from each other, and seemed to be inflamed with a massive quantity of inflammatory cells (Figures 8D,E).

**FIGURE 9:**
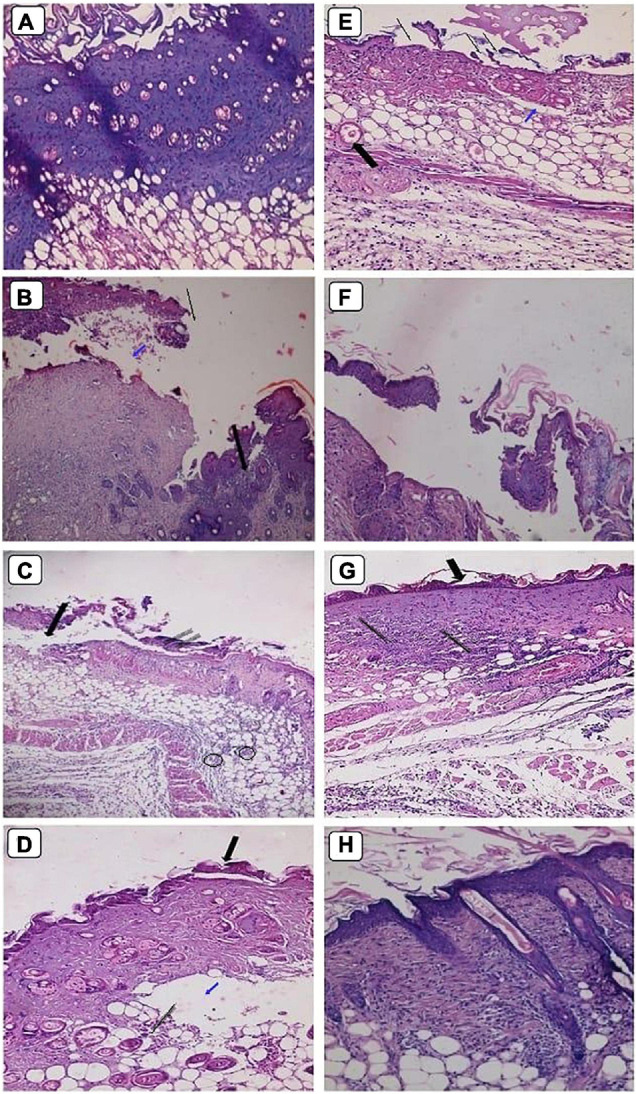
Histopathological examination of skin mice groups burned, infected and received different treatment during 1st stage **(A–D)**, and 2nd stage of treatment **(E–H)**. G4 skin treated with Ag-NPs gel **(A,E)**. G5 skin treated with neomycin gel **(B,F)**. G6 skin treated with neomycin-Ag-NPs gel **(C,G)**. G7 skin treated with neomycin-Ag-NPs spray **(D,H)**. Magnification power (200×). **(A)** Discontinuity of epithelial cells with vacuolation in the underlying epidermis. **(B)** Loss of epidermis (thin arrow), edema space with large lytic area (blue arrow), and massive infiltration with inflammatory cells (thick arrow). **(C)** Loss of epidermis layer underlying a massive infiltration with inflammatory cells (thin arrow), separation between dermis, and epidermis layers (thick arrow), and infiltration with inflammatory cells between the fat cells (circles). **(D)** Migrating epithelium cells (thick arrow) with the appearance of edema space (blue arrow) in the dermis layer embedded with mild infiltration (thin arrow). **(E)** Discontinuity of epidermis (thin arrow), edema space in the dermis layer (blue arrow), and congested blood capillary (thick arrow). **(F)** Separation between skin layers with edema space underlying the epithelium cells with massive infiltration. **(G)** Distortion of epidermis layer in few areas (thick arrow), and massive infiltration in the dermis layer (thin arrow). **(H)** Normal-looking skin with regular collagen distribution, normal-looking hair follicle.

On the other hand, the mice of G4 that burnt, infected with *P. aeruginosa* P_8_, and treated with Ag-NPs gel displayed some distortion and discontinuity in epithelial cells with inflammatory cell infiltration (Figure 9A). The 3-day follow-up of treatment with neomycin gel in G5 showed less healing activity where the epidermis layer was still completely lost with large lytic areas within the underlying epidermal layers (Figure 9B). This injury was also applied to the dermal layer with massive infiltration and the presence of rarified collagen (Figure 9B). When compared at the end of the treatment, the G4 skin was less affected than the untreated community with partial discontinuity in the epidermis (Figure 9E). G5 showed less affectivity where the epidermis and dermis layers tended to be differentiated by the presence of edema space and massive infiltration, which suggested that this formula was not successful in treating of burns (Figure 9F).

At the initial stage of treatment, G6 treated with neomycin-Ag-NPs gel displayed evidence of the appearance of migrating epithelium cells with only a slight infiltration of the underlying dermis layer (Figure 9C). Additionally, our findings revealed that in G7 treated with neomycin-Ag-NPs spray, marked healing signs have been found (Figure 9D). At the same time, the epidermis layer remains abnormal, and the dermis layer started to maintain its probable appearance with few inflammatory cells (Figure 9D). However, at the end of the treatment time, G6 seemed mildly disturbed with some distortion of the epidermis layer, but only in a few regions with the remaining signs of inflammatory cell inflammation (Figure 9G). Treatment of G7 with neomycin-Ag-NPs spray resulted in a significant healing impact by keeping the internal skin layers with much of their regular appearance with a regular distribution of collagen and the appearance of simply normal hair follicles, which indicated that this formula was the strongest one among the other formulations with the highest effectiveness in burn treatments (Figure 9H).

**FIGURE 10:**
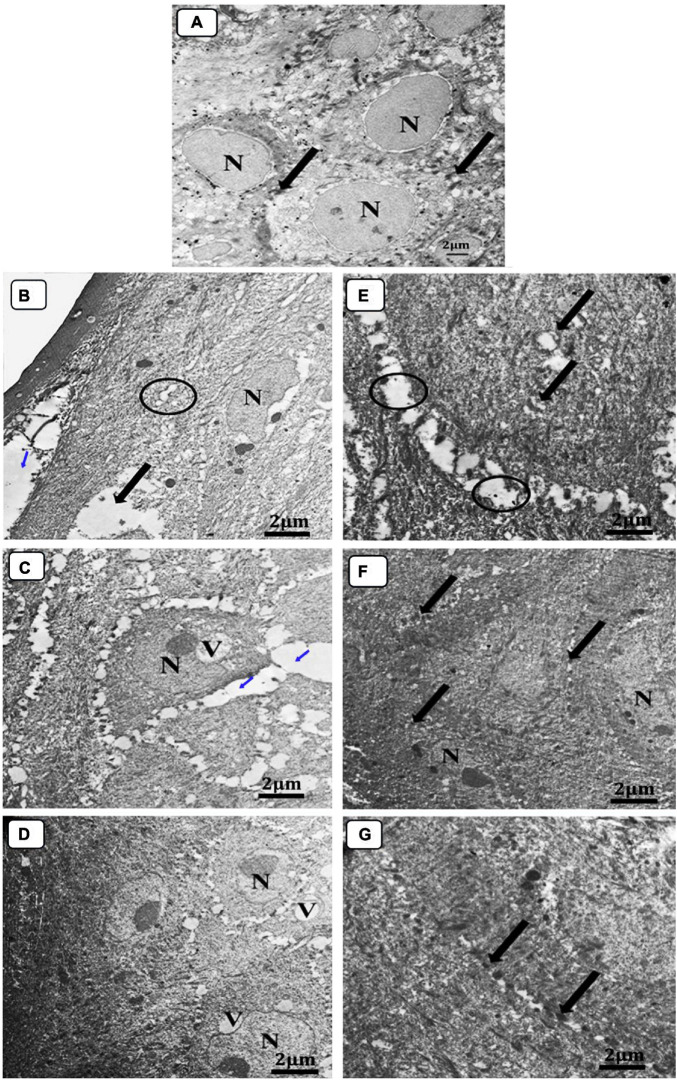
Electron micrograph of skin sections. **(A)** Normal group (negative control), **(B)** infected, and untreated (positive control), **(C)** infected, and treated with placebo base gel (positive control), **(D)** infected, and treated with Ag-NPs gel, **(E)** infected, and treated with neomycin gel, **(F)** infected, and treated with neomycin-Ag-NPs gel, and **(G)** infected, and treated with neomycin- Ag-NPs spray. **(A)** Normal epidermis layer in G1 normal oval nucleus (N) surrounded by homogenous cytoplasm with intact desmosomes and to no filaments (arrow). **(B)** Normal dermis layer, G2 separation between the upper layer of epidermis with widening in the intracellular spaces between the cells (blue arrow), disrupted desmosomes (circle), rarified cytoplasm (thick arrow), and irregular nucleus (N) surrounded by small lytic areas, **(C)** loss of desmosomes with large separation between the cells (blue arrow), degenerated mitochondria, and a large vacuole (V) that compresses the nucleus causing irregulation of the nucleus (N). **(D)** Start healing appearance in the superficial layer while the middle layer still shows irregular nucleus (N) and cytoplasmic vacuolation (V) with less intracellular spaces between cells most of desmosomes are complete and most of the mitochondria are probably normal. **(E)** Swollen degenerated mitochondria (thick arrow), rarified cytoplasm and disrupted of desmosomes (circle). **(F)** Mild irregular nucleus (N), most of the desmosomes are completely intact (thick arrow) and most of the cells are probably normal. **(G)** Most probably normal epidermis layer, and almost all desmosomes are intact (thick arrow).

The skin sections of mice in various treatment groups were evaluated by TEM relative to normal skin. The ultra-structures of the epidermis and dermis layers were studied, and the damage inside these layers has been established by comparison to differences in the features of the collagen fibers, nucleus, mitochondria, cytoplasm, as well as changes in the connective tissue between cells within the same layer or between two consecutive layers (Figure 10). The investigation of normal skin (G1) presented the normal appearance of an oval-shaped nucleus, normal mitochondria in the cytoplasm of the epidermis layer, and the desmosomes (molecular complexes of cell adhesion proteins and linking proteins specialized for cell-to-cell adhesion) between the cells were intact without any separations (Figure 10A). In both groups, G2 and G3, the epidermis and dermis layers were seriously damaged and separated from each other (Figures 10B,C). The presence of an abnormal nucleus, degenerated mitochondria, and rarified cytoplasm, as well as the absence of cell-to-cell connections due to desmosome disruption and significant lysis in the cells, was also observed, indicating the extent of the burn effect on the skin layers (Figures 10B,C). Although the skin was still damaged in G4, the subsequent damage was less than that incurred by neomycin gel therapy (G5) (Figures 10D,E). The desmosomes were more disrupted, and the skin layers were more separated with wider intracellular spaces and vacuolation in the case of neomycin treatment (Figure 10D). While Ag-NPs gel treatment resulted in the denatured nucleus and cytoplasmic vacuolation in the epidermis layer and collagen fibers disorganization in the dermis, there was enhancing healing activity observed by the less intracellular spaces between cells where most of the desmosomes appeared completed, and the majority of mitochondria were probably normal (Figure 10E). Neomycin and Ag-NPs as a gel (G6) in the skin layers have resulted in better healing effects relative to other previous groups (Figure 10F). Even though the nucleus of certain cells looked irregular and there was evidence of the presence of inflammatory cells, the intracellular spaces between the cells decreased, most desmosomes were connected, and most of the inflammatory cells degenerated.

Interestingly, with neomycin-Ag-NPs spray (G7), improved healing activity was obtained. Skin layers were most definitely natural. Many cells tended to be bound to desmosomes with the presence of a normal nucleus, mitochondria, and cytoplasm, as well as collagen in the dermis layer, which retains its organization (Figure 10G).

**FIGURE 11:**
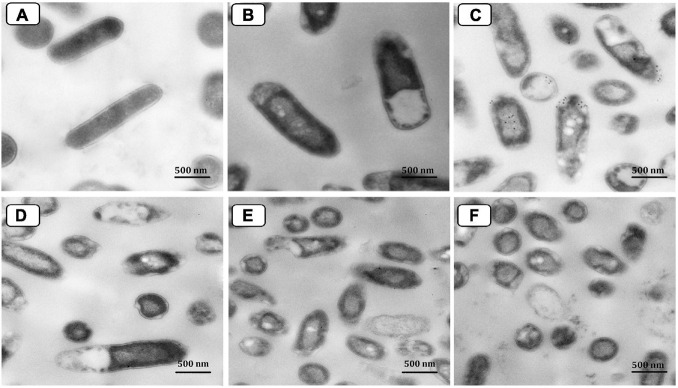
Transmission electron micrograph of *P. aeruginosa* P_8_ cells under different treatments. Untreated cells **(A)**. Cells treated with neomycin only **(B)**. Cells treated with Ag-NPs only **(C)**. Cells treated with neomycin-Ag-NPs combination **(D–F)**.

Transmission electron micrograph of *P. aeruginosa* P_8_ cells under different treatments was also performed (Figure 11). With a smooth and intact cell wall and cell membrane, the morphological features of the control (untreated) cells appeared regular (Figure 11A). However, the TEM investigation of the neomycin-treated cells showed only a minor effect suggested by protoplasmic shrinkage, whereas the cell wall remained intact in most cells (Figure 11B). Moreover, the resulting damage from Ag-NPs treatment of cells surpassed that caused by neomycin (Figure 11C). The membrane of treated cells was damaged with the presence of many pits and gaps appeared in the cell walls; Ag-NPs appeared accumulated on the surface of the cells with partial lysis in the cell walls beside the presence of some vacuoles within the cells. Also, cell treatment with the combination of neomycin-Ag-NPs had resulted in significant damage to those cells where the cell walls were completely lysed. The cells were ruptured with releasing cell components outside the cells (Figures 11D–F).

## Discussion

Today, the enormous rise in bacterial tolerance to antimicrobials is becoming a major concern for all health care organizations to minimize the production of newly resistant isolates by continuous testing for alternatives to traditional antibiotics used (El-Adawi et al., 2013; Khalil and Sonbol, 2014; El- Shouny et al., 2016; Mohanty et al., 2020). Herein, the present research focused on applying nanotechnology to the alteration of the action of certain antibiotics against MDR bacteria, causing burn infections by combining these antibiotics with certain metal nanoparticles. In the same background, Mohamed (2016) observed that the most widespread bacteria recovered from burned patients were *P. aeruginosa* (46%). Therefore, *P. aeruginosa* isolates were chosen for this analysis. Their susceptibility testing revealed a strong degree of observable antimicrobial tolerance to various antibiotics in particular; Amoxicillin, Amoxicillin/Clavulanic acid, Cefepime, and Tetracycline. Khalil et al. (2015b) recorded that among 104 *P. aeruginosa* isolates recovered from patients admitted to Tanta University Hospitals, 100% of isolates were resistant to Ampicillin, Amoxicillin, Amoxicillin/Clavulanic acid, Cefepime, Ceftriaxone, Ceftizoxime, Kanamycin. Also, Sonbol et al. (2015) confirmed the existence of β-lactamase and extended-spectrum β-lactamase genes in certain multi-drug resistant *P. aeruginosa* isolates. Nanotechnology and medical applications of some metal nanoparticles have recently increased their interest, especially in their use as an antimicrobial against MDR isolates (Sánchez-López et al., 2020). Due to their unique physical and chemical properties, such as their wide surface area to volume ratio, nanomaterial has been proposed to play a promising function, enabling them to interact with microbial membranes (Beyth et al., 2015).

According to the findings, the combination of Ag-NPs significantly improved the antibacterial efficacy of most of the antibiotics tested, as evidenced by a rise of up to eight times the inhibition zone diameters. Related research results were also reported by Panáček et al. (2016). Brown et al. (2012) demonstrated synergism of Ag-NPs and even gold nanoparticles complexed with ampicillin, against multi-resistant strains such as MDR isolates of *P. aeruginosa*, *Enterobacter aerogenes*, and MRSA. Kumar et al. (2016) reported that tetracycline conjugated Ag-NPs increased the antibacterial action of tetracycline due to enhanced accumulation of the Ag^+^ around the bacterial cell membranes. Similarly, the synergistic antimicrobial activity of Ag-NPs with chloramphenicol and gentamicin against MDR *Enterococcus faecalis* compared to antibiotics alone (Katva et al., 2018). It was proposed that Ag-NPs may collaborate with cell wall synthesis to prevent antibiotics, encourage extensive damage to the cell wall, and also facilitates the transfer of hydrophilic antibiotics to the cell surface, in addition to the capacity of Ag-NPs to improve the cell membrane permeability, thereby allowing antibiotics to enter the cells more easily (Deng et al., 2016).

The increased usage of the nanoparticle has also raised concerns about the emergence of AgNPs-resistant bacteria (Panáček et al., 2018; Hosny et al., 2019; Valentin et al., 2020; McNeilly et al., 2021). *P. aeruginosa* P16 was resistant to all antibiotics, AgNP, and antibiotic-AgNP combinations, according to obtained results. In the same context, Salomoni et al. (2017) discovered that *P. aeruginosa* P.a.2 from a hospital was resistant to all antibiotics and AgNPs tested. This is concerning since the growing indiscriminate use of Ag-NPs may encourage the development of silver resistance in this species, as has happened with antibiotics. It is possible that exposing bacteria to harmful heavy metals such as silver may cause the development of antibiotic resistance through the co-selection process. Bacterial resistance to silver, particularly Ag+, has been widely documented, while evidence of AgNPs-specific resistance is still developing. Until far, research has revealed the existence of both external and endogenous genetic determinants of Ag+ resistance, which are believed to be relevant to AgNPs as well (Graves et al., 2015; Panáček et al., 2018;Valentin et al., 2020).

Based on our finding from the marked *in vitro* antibacterial activity of Ag-NPs, selected for use in the *in vivo* experiment to treat bacterially infected burns induced in Swiss albino mice as alternatives to widely recognized silver-containing drugs such as AgNO_3_ or SSD. Various topical antimicrobial formulations focused on Ag-NPs were *in vivo* reviewed as delivery systems to treat induced burns infected with the selected bacteria. Neomycin antibiotic was chosen as a controlled drug where its MIC was calculated in the presence and absence of sub-inhibiting concentration of Ag-NPs against two MDR *P. aeruginosa* P_8_ and P_14_ isolates. Maximum synergism was observed by neomycin Ag-NPs combination against P_8_ isolate, with an eightfold decrease in neomycin MIC values reported. As a consequence, isolate P_8_ was selected for the *in vivo* trials. Mice have been exposed to the third degree of burn and were purposely infected with isolate P_8_. To ensure the safety of the formulated drug formulations for topical usage, these formulations were firstly applied directly on mice skin (G8) for a week before the experiment. It was found that not all the drug formulas under investigation culminated in any detectable signs of toxicity. In this context, Ag-NPs recorded no cytotoxic effects on human cells at concentrations below 30 mg/l (Krajewski et al., 2013; Munger et al., 2014). Also, the hemolytic activity of Ag-NPs on human erythrocytes was examined by Katva et al. (2018) and recorded that 10–100 μg/ml concentration of Ag-NPs didn’t induce any hemolysis of blood cells, including erythrocytes. This is believed to be a product of the strong surface-to-volume ratio of Ag-NPs, which helps the particles to stay effective even at a very low concentration and thus minimizes the risk of tissue toxicity (Adhya et al., 2015).

To identify the most effective formula, G7 treated with neomycin-Ag-NPs spray showed the greatest decrease in the number of bacterial counts on 3rd day of treatment with a maximum inhibition of bacterial growth at the end of the treatment. Interestingly, the spray mixture also showed the highest healing activity and the highest percentage of wound contraction among the other treatments. The healing activity of Ag-NPs have been extensively studied before (Vaidyanathan et al., 2009; Sundaramoorthi et al., 2011; Adhya et al., 2015; Ahmadi and Adibhesami, 2017).

Transmission electron microscope photographs revealed that G7 (treated with neomycin-Ag-NPs spray) showed the greatest signs of healing and lowest inflammation, in which the skin’s inner layers retained most of their normal appearance. Ag-NPs’ anti-inflammatory role in burns and other wounds cure is due to reducing inflammatory cell infiltration and inhibiting the development of inflammatory cytokines (Baharara et al., 2017). The improved performance of the combination being tested in spray formula as shown by either comparatively higher antibacterial activity or better healing activity than the same combination in gel formula may be linked or even contingent upon the combination’s physical design itself. Therefore, the mobility of nanoparticles and antibiotics in spray form would be easier; they would be released from spray formula easier and faster than gel due to the diffusion properties of colloidal nanoparticles by gel (Panáček et al., 2016).

Transmission electron microscope examined cells microscopically pre and post-treatment with Ag-NPs alone, with neomycin alone, or with a combination of both agents for 12 h compared to control (untreated) cells. Neomycin-treated cells were found to be significantly compromised with only protoplasmic shrinkage, but the cells appeared comparatively less damaged than those treated with Ag-NPs. This observation may explain the relatively lower antibacterial activity of neomycin *in vitro* compared to the Ag-NPs. Sindhu et al. (2013) previously studied the potent bactericidal behavior of Ag-NPs. It was thought to depend on the release of cationic silver ions (Ag^+^) as nanoparticles dissolve in water or penetrate cells. These ions are suggested to cross the bacteria’s hydrophobic cell membranes and thus enter the cells *via* an association with transmembrane proteins that normally function to hold ions other than silver.

Generally, It was reported that the antibacterial mechanisms of Ag-NPs cab be attributed to the release of Ag ions and NPs deposition inside the bacterial cells. Such mechanisms mainly involve damaging the cellular membrane, generation of Reactive Oxygen Species (ROS), disruption of energy metabolism, and gene transcription (Li et al., 2010; McShan et al., 2014). Yan et al. (2018) reported that membrane proteins and oxidative stress are the main antibacterial mechanisms induced by Ag-NPs against *P. aeruginosa*. On the other hand, several membrane proteins whose key role was antibiotic tolerance, ion binding, pore-forming, membrane stabilization, and flagellum assembly were up-or down-regulated by the Ag-NPs. In that context, three outer membrane porins (OprC, OprD, and OprH) associated with the transport of antibiotics, amino acids, and peptides were significantly affected by Ag-NPs Yan et al. (2018). Also, the metal transporters, namely CcoO1/CcoO2 (Fe), OprC (Cu), and PA0372 (Zn), were all suppressed in *P. aeruginosa* upon exposure to Ag-NPs, which may assist with the transport of Ag ions and Ag-NPs into the bacterial cell through the *trans-*membrane pores (Yan et al., 2018).

Furthermore, Kirov (2003) reported that the regulated flagellin proteins (pilX, pilP, FliN, and FlgE) are rich in lipids and play an important role in biofilm formation, adhesion, and motor activities. It was further suggested that the large surface area of nanoparticles could facilitate their interaction with active antibiotic groups, such as hydroxyl and amine groups, resulting in the conjugation of both molecules (antibiotic-AgNP complexes) and thus increase the antibiotic concentration at the injection site (Sindhu et al., 2013; Kumar et al., 2016; Dixit et al., 2017). Another potential mechanism that may lead to increase antibiotic activity by the combination of Ag-NPs is proposed to be the inhibition of some bacterial enzymes that are responsible for the tolerance of bacteria to antibiotics (Panáček et al., 2016). For example, β-lactamase, carbapenemase, and other enzymes may be coated or attached to surfaces of nanoparticles resulting in a modification of their structure resulting in their inactivation. As depicted in TEM photographs, Ag-NPs were found to be accumulated on the surface of the cells with partial lysis in the cell walls beside the presence of some vacuoles within the cells. Our findings agree with those reported previously (Raffi et al., 2008; Fabrega et al., 2009; Mirzajani et al., 2011). Therefore, Ag-NPs were probably attached to the surface of the cell membranes and affected the expression of related proteins. Oxidative stress that results from the increased ROS production has also been reported as a major mechanism of Ag-NPs-induced toxicity in human cells since oxidative reductases, and superoxide dismutases are involved in intracellular oxidative damage caused by metal ions (AshaRani et al., 2009; Oh et al., 2016). Indeed, *in vivo* histopathological results exhibited that Ag-NPs may be effective as promising antimicrobial agents against MDR *P. aeruginosa* inhabiting burn wound infections. However, further studies based on proteomic analysis are required to identify many membrane proteins that can be attributed to the toxicity of *P. aeruginosa* by Ag-NPs. Moreover, more investigations are needed on human volunteers with skin burns to confirm the efficacy of Ag-NPs alone or in combination with an antibiotic(s) as novel antibacterial agents in wound healing, especially after the success of Ag-NPs and the combination of neomycin-Ag-NPs in the treatment of burn wounds of the experimental animal models.

## Conclusion

The incorporation of Ag-NPs in various pharmaceutical formulas for the *in vivo* treatment of bacterially contaminated burns revealed an interesting antibacterial and healing activity, especially when combined with antibiotics and topically applied in a spray form. Nanomaterials are therefore especially promising in burning infections as an antibacterial complement to antibiotics. They should be of greater interest because they can fill the gaps where antibiotics frequently fail to combat multidrug-resistant pathogens.

## Data Availability Statement

The raw data supporting the conclusions of this article will be made available by the authors, without undue reservation.

## Ethics Statement

The studies involving human participants were reviewed and approved by the Faculty of Medicine, Tanta University, Tanta University Hospital Research Ethical Committee Centers. The patients/participants provided their written informed consent to participate in this study. The animal study was reviewed and approved by the ethical guidelines approved by the Animal Ethics Committee Guide Lines of Tanta University. Written informed consent was obtained from the owners for the participation of their animals in this study.

## Author Contributions

MK: conceptualization, methodology, formal analysis, data curation, and writing—review and editing. GE: methodology and investigation. FS: conceptualization, validation, and visualization. NA: investigation and formal analysis. PA: methodology and preparation. SA: formal analysis, data curation, and writing—review and editing. All authors contributed to the article and approved the submitted version.

## Conflict of Interest

The authors declare that the research was conducted in the absence of any commercial or financial relationships that could be construed as a potential conflict of interest.

## Publisher’s Note

All claims expressed in this article are solely those of the authors and do not necessarily represent those of their affiliated organizations, or those of the publisher, the editors and the reviewers. Any product that may be evaluated in this article, or claim that may be made by its manufacturer, is not guaranteed or endorsed by the publisher.
