# Supplementary material for: Enhanced Efficacy of Some Antibiotics in Presence of Silver Nanoparticles Against Multidrug Resistant Pseudomonas aeruginosa Recovered From Burn Wound Infections
Source: Front Microbiol. 2021 Sep 20;12:648560. doi: 10.3389/fmicb.2021.648560 (PMC8488261; doi:10.3389/fmicb.2021.648560)
Supplement: Supplementary file 1 [file Table_1.DOCX]

**Table S1**

The composition of the prepared drug formulations.

| Formulation | Ingredients | | | |
| --- | --- | --- | --- | --- |
|  | Neomycin (g) | Methylcellulose (g) | Ag-NPs (1.7 µg/ml) | Deionized dist. H_2_O (ml) |
| Ag-NPs gel | - | 4.5 | 100 | - |
| Neomycin gel | 0.5 | 4.5 | - | 100 |
| Neomycin-Ag-NPs gel | 0.5 | 4.5 | 100 | - |
| Neomycin-Ag-NPs spray | 0.5 | - | 100 | - |
| Placebo gel | - | 4.5 | - | 100 |
